# Advances in Drug Design Based on the Amino Acid Approach: Taurine Analogues for the Treatment of CNS Diseases

**DOI:** 10.3390/ph5101128

**Published:** 2012-10-23

**Authors:** Man Chin Chung, Pedro Malatesta, Priscila Longhin Bosquesi, Paulo Renato Yamasaki, Jean Leandro dos Santos, Ednir Oliveira Vizioli

**Affiliations:** Lapdesf-Laboratory of Drug Design, School of Pharmaceutical Sciences, University of São Paulo State (UNESP), Rodovia Araraquara-Jaú Km1, CEP 14.801-902, Araraquara, SP, Brazil; Email: pedromalat@hotmail.com (P.M.); bosquesi@fcfar.unesp.br (P.L.B.); paulorenatoyamasaki@gmail.com (P.R.Y.); santosjl@fcfar.unesp.br (J.L.S.); ednirvizioli@yahoo.com.br (E.O.V.)

**Keywords:** amino acid, CNS, taurine, analogs

## Abstract

Amino acids are well known to be an important class of compounds for the maintenance of body homeostasis and their deficit, even for the polar neuroactive aminoacids, can be controlled by supplementation. However, for the amino acid taurine (2-aminoethanesulfonic acid) this is not true. Due its special physicochemical properties, taurine is unable to cross the blood-brain barrier. In addition of injured taurine transport systems under pathological conditions, CNS supplementation of taurine is almost null. Taurine is a potent antioxidant and anti-inflammatory semi-essential amino acid extensively involved in neurological activities, acting as neurotrophic factor, binding to GABA A/glycine receptors and blocking the excitotoxicity glutamate-induced pathway leading to be a neuroprotective effect and neuromodulation. Taurine deficits have been implicated in several CNS diseases, such as Alzheimer’s, Parkinson’s, epilepsy and in the damage of retinal neurons. This review describes the CNS physiological functions of taurine and the development of new derivatives based on its structure useful in CNS disease treatment.

## 1. Introduction

Amino acids (AA) are an important class of cell signaling molecules, involved in the regulation of gene expression and the protein phosphorylation cascade. They are also precursors of hormone synthesis and low-molecular nitrogenous substances [1]. Taurine (2-aminoethanesulfonic acid) is a β-AA and is the most abundant amino acid in mammals, being widely distributed in the CNS occupying the second place after glutamate in relation of its concentration, which differs depending on the regions of brain activity and animal species studied [2] and presenting different functions, which have been studied for their potential in neurology as a trophic factor in brain development, in regulating calcium transport, in the integrity of the eardrum, as osmoregulator, neurotransmitter, neuromodulator and for its neuroprotective action [3].

Taurine was first isolated from ox bile for over 150 years, it was considered a sulfur metabolism end product with no biological activity [4]. Recently, several researchers have reported the physiological function of taurine in the liver, kidney, heart, pancreas, retina and brain and the fact that its depletion is associated a several disease conditions such as diabetes [5,6,7], Parkinson’s [8], Alzheimer’s [9,10], cardiovascular diseases [11,12,13], and neuronal damages in the retina [14].

**Scheme 1:**
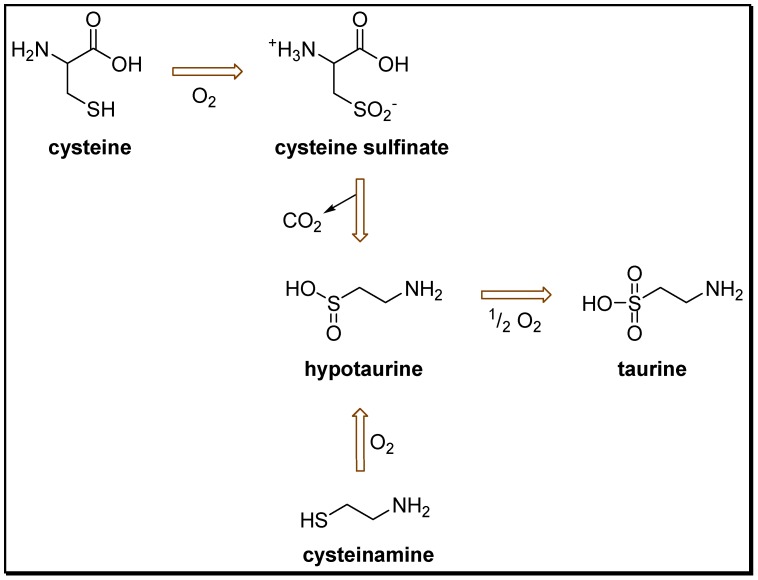
Biosynthesis of taurine.

Even though taurine is the most abundant free AA in mammals, man and cats lack the ability to synthesize taurine in sufficient quantities [15]. The biosynthesis takes place in the liver and starts from methionine, through cysteine, leading to cysteine-sulfonic acid which is converted to hypotaurine and taurine (Scheme 1). It was also demonstrated that taurine biosynthesis in the hippocampus and cerebellum ocurrs through the conversion of the amino acid cysteine by the sulfinic acid decarboxylase enzyme (CAD/CSAD and taurine-synthase), [16,17].

Despite the fact taurine is used in the market in energetic drinks (mistakenly known by the population as a stimulant agent), the neuroinhibitory effect of taurine in the central nervous system (CNS) was acknowledged in reports as early as the decade of the 19?60s. Over the following decade, several studies were carried out in order to gain a deeper insight into this function, and the mechanism behind it [18]. 

Taurine acts in these areas by binding in specific Tau receptors (TauR) promoting neuronal hyperpolarization via opening of chloride channels [19,20], and producing depressor activity by specific action via GABA A, GABA B and/or the glycine receptor [21].

The action of taurine in GABA_A_ receptors counteracts the seizures produced by picrotoxin, a GABA_A_ antagonist, increasing the latency of such seizures both acutely and chronically [22]. Besides the GABA_A_ agonistic activity, taurine also increases GABA levels, enhancing the production of the two isoforms of the glutamic acid decarboxylase (GAD 65 and 67), involved in the GABA synthesis [23].

Taurine is involved in the modulation of the excitotoxicity produced by glutamate, through the regulation of calcium homeostasis. This effect is related to a neuroprotective action. It is known that glutamate has affinity for *N*-methyl-D-aspartate (NMDA) receptors, through which the calcium influx occurs. This event activates a cyclic guanosine monophosphate (cGMP)-mediated pathway, culminating in the activation of the protein kinase C (PKC), responsible for the reduction of the magnesium block of NMDA channels, increasing the calcium influx and excitotoxicity [24].

In a stress situation, the neurotoxicity trigger is activated by an excess of glutamate delivery and taurine is quickly evocated for delivery in this situation [25]. Evidence of the taurine neuroprotective effects from β-amyloid action and glutamate receptor agonists involves the neutralization of the NMDA receptors, reduction of the glutamate delivery and the NO superproduction via GABA A activation. In fact, this strongly suggests the taurine prevention in Alzheimer´s disease and other neurological disorders [26]. Similar results were observed in the neuroprotection by taurine against the excess of ammonia and cerebral edema [27,28].

Because taurine has a sulfonic acid instead a carboxylic acid group it presents unique physical properties in comparison to other neuroactive AAs that make it difficult to cross the blood-brain barrier (BBB). In addition, it’s a monobasic acid, with very low solubility in water (10.48 g/100 mL at 25 °C); the pKa value is 1.5 (more acidic than glycine, aspartic acid, β-alanine and GABA). The pKb value is 8.82 (less basic than glycine, β-alanine and GABA). The low passive diffusion of taurine occurs because of its cyclic conformational form with intra-molecular hydrogen bonding [15]. 

The concentrations of taurine in the CNS are dependent on feeding and a complex transport across TauT specific complex systems at the blood brain barrier (BBB) and it may be involved in the maintenance of taurine levels in the brain in order to protect it against CNS damage [29].

It was reported that the TauT at the BBB was reduced in spontaneously hypertensive rats in comparison with normotensive rats [30]. Also, in other disease conditions or oxidative stress processes, CNS transport of taurine at the BBB fails [29]. Taurine levels were increased in the brain interstitial fluid in ischemia [31] and in the acute phase of Parkinson’s [32]. However, in a chronic situation of the same disease (Parkinson’s), taurine levels are low [8]. In the acute phase of the diseases, taurine is available to protect CNS, but if the BBB taurine transport also fails and because taurine cannot cross the BBB by itself, there is not a sufficient concentration for neuroprotection and then the disease evolves to the chronic phase.

In addition to the physicochemical properties of taurine that promote low passive diffusion through the membranes, and low gastro-intestinal absorption, it is very interesting to plan new lipophilic taurine derivatives that can cross the BBB in disease conditions and or/increase binding receptors.

## 2. Anticonvulsant Taurine Analogues

**Figure 1:**
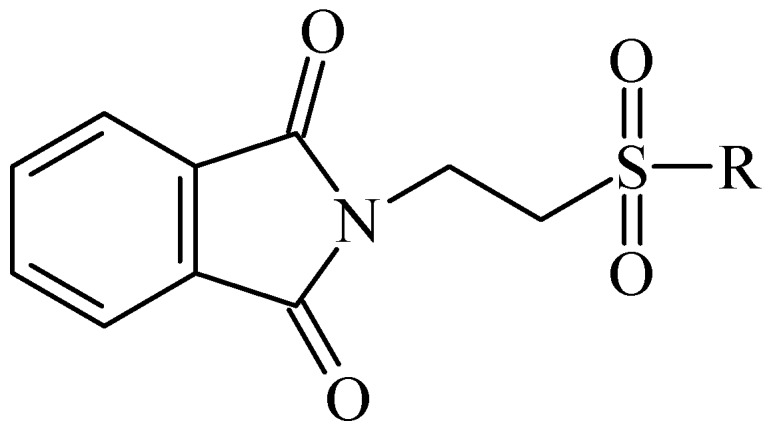
General structure of phthalimidoetanosulfonamide derivatives.

In 1983, Lindén and co-workers [33] synthesized 2-phthalimidosulfonamide derivatives of taurine (Figure 1) and tested their anticonvulsant activity. The structure-activity relationship study showed that the two-carbon chain of the taurine molecule is essential for a better activity of these derivatives. Furthermore, it was also noted that substitutions in the terminal sulphonamide moiety increased the lipophilicity of the molecules, thus facilitating the drugs’ access into the brain. However, it was observed that the activity decreased with voluminous groups attached to the sulphonamide moiety.

The anticonvulsant activity in the maximum electroshock seizure (MES) model and in the subcutaneous pentetetrazole seizure threshold (PST) model were effective with unsubstituted amide, methylamide, dimethylamide, ethylamide and isopropylamide derivatives and the efficacy was almost equal. *N*-Propylamide and *N*-butylamide were also active, but less potent, No effect were observed in acetamide, pyrrolinedide, piperidide, cyclohexylamide, benzylamide, methylbenzylamide and pyridylamide derivatives. The *N*-isopropyl derivative (named taltrimide) is now commercially but it is not approved for therapeutic use as the anticonvulsive effects of taltrimide observed in animal experiments were not confirmed in clinical trials. In contrast, the seizures increased statistically significantly during taltrimide treatment, suggesting a proconvulsant effect of taltrimide in humans and the reason for this remains obscure [34].

**Figure 2:**
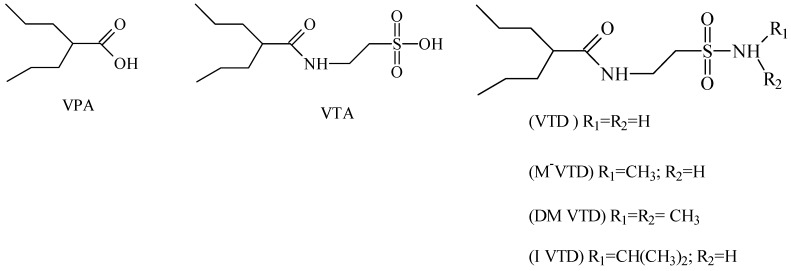
Valproyltaurine derivatives.

Isoherranen and co-workers [35] synthesized novel valproyltaurinamide derivatives (Figure 2), that could act not only as mutual prodrugs of valproic acid (VPA) and taurine, but also as a hybrid one. The purpose of this work was to obtain better a valproic acid, an antiepileptic drug useful against a variety of types of epileptic seizures and compounds devoid of teratogenic effects. Three compounds showed good profile (VTD > I-VTD and DM-VTD) in preventing tonic extension, clonus and wild running.

In the pharmacokinetics assay, a good correlation with the brain metabolite *N*-alkyl-VTD and its anticonvulsant activity was observed, but no correlation was found with log P value and teratogenic potency. Neural tube defects were observed in the order VTA > DM-VTD > I-VTD > VTD. The compound M-VTD had a very low teratogenic potential (1% of the live born fetuses, not significant, *P* > 0.05).

Based on the results with valproyltaurinamide derivatives and the knowledge of that anilide groups, with small substituents on the *N*-phenyl ring, are known to produce potent anticonvulsants, in 2007 Akgul and co-workers, repeated the Lindén *et al*. research [33] and obtained 15 new 2-phtalimidoethanesulfonamide derivatives with phenyl groups attached to the sulphonamide moiety The preliminary screening results indicated that the exchange of the *N*-isopropyl moiety for an *N*-phenyl ring in the taltrimide molecule abolished the anticonvulsant activity. However, introducing certain substituents, such as nitro, methyl, and chloro, into the *N*-phenyl ring lead to more active compounds in the MES test in comparison to the unsubstituted derivatives (Table 1). In the rotarod test for neurotoxicity effects were observed with a methyl substituent in the *N*-phenyl ring [36].

**Table 1:** Best phenyl phtalimidoethanesulfonamide derivatives in MES test after 0.5 and 4 h of administration and the compounds’ neurotoxicity [36]. 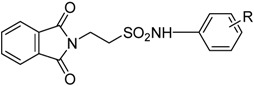

	Time of the test after administration of the drug	
	0.5 h	4 h
Best activity in MES test	(I) R = 3-NO_2_	(III) R = 2-CH_3_
	(II) R = 2-Cl	(IV) R = 2-CH(CH_3_)_2_
		(V) R = 4-NO_2_
Higher neurotoxicity	(VI) R = 3-CH_3_	(VII) R = 2-CH_3_

**Figure 3:**
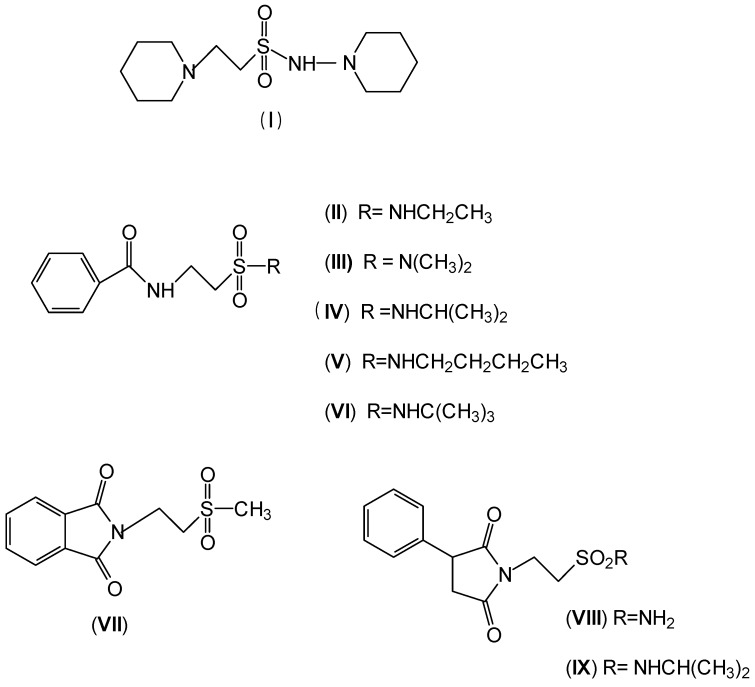
Structures of the compounds with anticonvulsant activity [37].

Oja and co-workers obtained 23 taurine derivatives substituted at the amine and sulfonic acid group. Figure 3 shows nine of the active compounds: piperidino (VIII) benzamido (IX-XIII), phthalimido (XIV) and phenylsuccinimido (XV-XVI) derivatives. Compound VIII showed the best activity when tested in rotarod method (used to assess motor coordination and balance in rodents) than other compounds and greater than valproate and diazepam when its effects were calculated from both the MES and PST test and compound I showed toxicity [37].

## 3. Alteration in Temperature Activity

**Figure 4:**
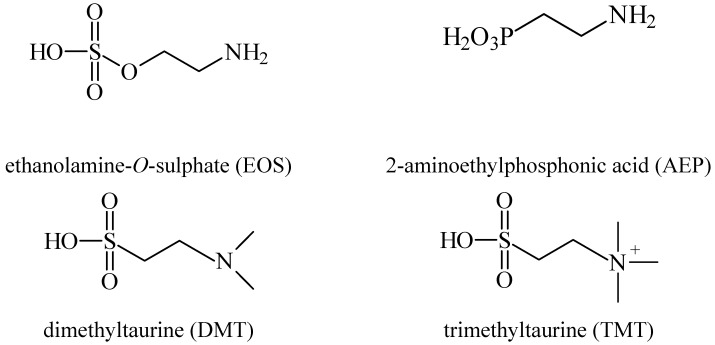
Structures of ethanolamine-*O*-sulphate (EOS), 2-aminoethylphosphonic acid (AEP), dimethyltaurine (DMT) and trimethyltaurine (TMT) with GABA_A_ and GABA_B_ antagonism.

Besides the anticonvulsant activity, agonistic binding of taurine to GABA sites is also known to cause hypothermia. This feature, whereby many taurine derivatives such as ethanolamine-*O*-sulphate (EOS), 2-aminoethylphosphonic acid (AEP), dimethyltaurine (DMT) and trimethyltaurine (TMT) (Figure 4) induced hyperthermia through the antagonism of GABA_A_ and GABA_B _was studied by Frosini *et al.* [38].

**Figure 5:**
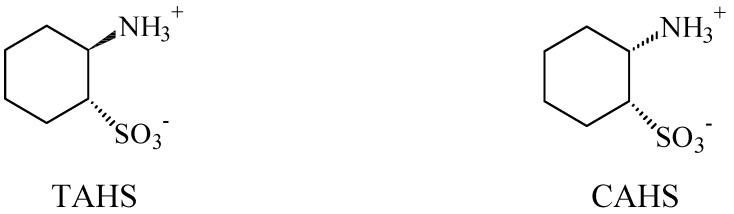
Structures of the derivatives *trans-* (TAHS) and *cis*-aminocyclohexanesulfonic acid (CAHS).

Frosini *et al.* explained why some taurine derivatives produce hyperthermia, as they bind to the taurine receptor without activating it, thus constraining taurine receptor agonists to activate the receptor to induce dissipation of body heat [39]. Furthermore, the study mentioned that the taurine receptor has a conformation which only allows the interaction of derivatives with the two functional groups in a axial-equatorial position. This explains why a derivative such as *cis*-amino-cyclohexaenesulfonic acid (CAHS) induces hypothermia whereas its *trans-*derivative (TAHS) is inactive (Figure 5). The ring of the latter is repulsed by the hydrophilic area of the taurine receptor.

## 4. Prevention of Alzheimer’s and Neuroprotection

**Figure 6:**
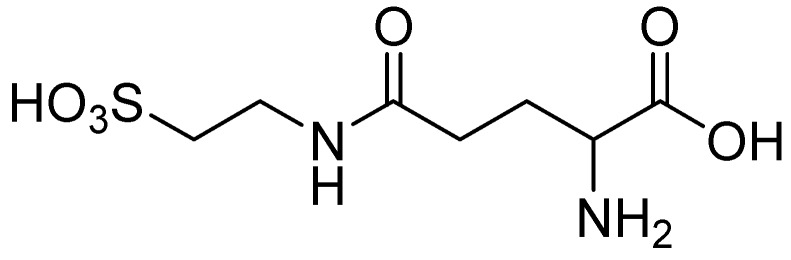
Structure of γ-L-glutamyltaurine.

Varga and co-workers proposed γ-L-glutamyltaurine (Figure 6) as an endogenous modulator in the excitatory aminoacidergic neurotransmission process inhibiting the glutamate-evoked increase in free intracellular Ca^2+^ and the kainate-activated formation of cGMP in cerebellar slices [40]. Studies also showed that γ-L-glutamyltaurine could prevent the genotoxic action of mitomycin C (MMC) in rat bone marrow cells using the micronucleus test [41]. However, this compound lacks good properties to be used for CNS diseases.

**Figure 7:**
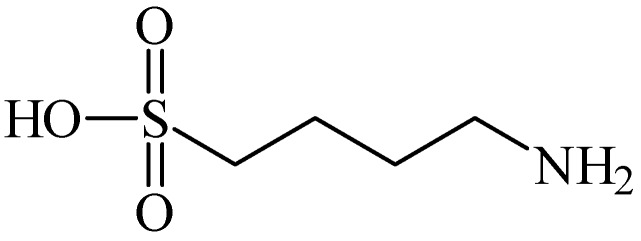
Structure of homotaurine (tamiprosate, Alzehmed™).

Addition of a carbon atom to the taurine molecule resulted in a new compound named homotaurine and it was first patented by Abbott Laboratories, in 1965 [42] (tramiprosate, Figure 7) as a promising drug for Alzheimer’s treatment named Alzhemed™. It binds preferentially to soluble Aβ peptide and maintains Aβ in a non-fibrillar form, thereby inhibiting amyloid formation and deposition. [43]. It was entered phase III clinical trials but failed to demonstrate efficacy in long-term clinical testing of cognitive improvement [44].

**Figure 8:**
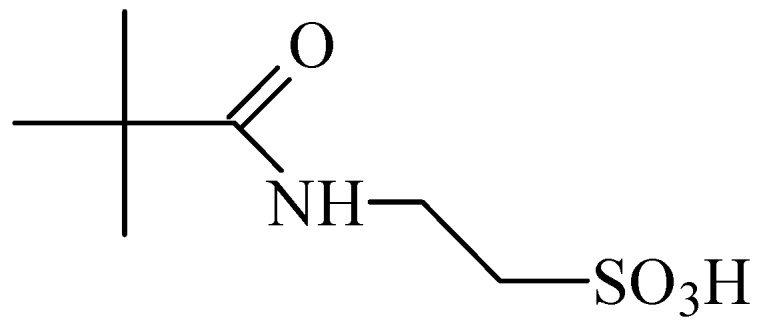
Structure of *N*-pivaloyltaurine.

Homotaurine was also related to elevation of striatal dopamine, in a manner independent of impulse flow or exocytosis. Differently, the taurine-evoked increase in striatal dopamine was impulse-flow dependent [45]. *N*-Pivaloyltaurine (Figure 8) is a prodrug of taurine that is slightly converted to taurine in the brain and shown to perform the same as taurine in striatal dopamine production [46].

**Figure 9:**
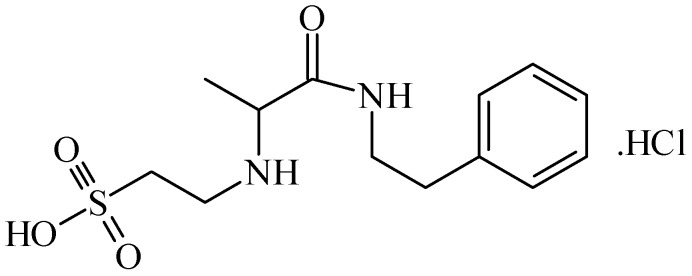
Structure of *N*-isopropylamine-2-(1-phenylethyl)aminoethanosulfonic acid hydrochloride (TAU-15, taurepar).

In a cerebral ischemia study, conducted by Sapronov *et al.*, the substance *N*-isopropylamide-2-(1-phenylethyl)aminoethanesulfonic acid hydrochloride (TAU-15, taurepar, Figure 9), was also demonstrated to possess neuroprotective effects [47]. In this study, where a brain ischemia situation was simulated through the occlusion of the common carotid arteries, TAU-15 presented a variety of effects: It activated the aerobic oxidation of carbohydrates, improving the energy metabolism; inhibited the hyperactivation of lipid peroxidation under ischemia conditions; restored the antioxidant system, regulating the generation of free radicals; and increase animal lifetime by 40%. In addition to the neuroprotective properties of TAU-15, it is claimed that besides the aforementioned actions, taurepar also has a positive effect in spinal cord compression [48].

**Figure 10:**
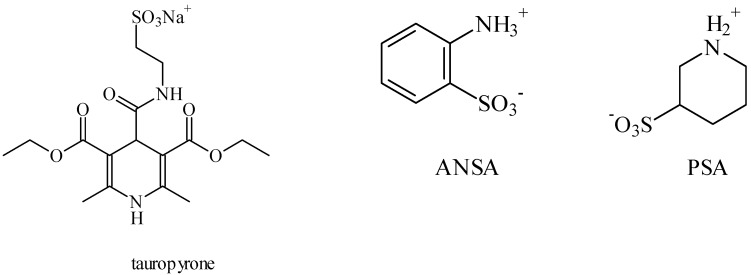
Structures of tauropyrone, piperidine 3-sulfinic acid (PSA), aniline 2-sulfinic acid (ANSA).

Tauropyrone (Figure 10) has also been proven to be a neuroprotective agent, without anticonvulsant activity [49]. According to Ward *et al.*, it was able to prevent the dopamine oxidation through the 6-OH-dopamine pathway, involved in Parkinson’s disease. Furthermore, unlike taurine, it does not depend on the taurine transporter to exert its functions, as it is more lipophilic. In another report, tauropyrone was claimed to prevent the oxygen-glucose deprivation (OGD) cell-damage, attenuating the production of lactate dehydrogenase, and the OGD-stimulated excitotoxicity by means of glutamate release [50]. In more recent research, the OGD model was used to evaluate taurine analogues that are GABA-T inhibitors. Piperidine 3-sulfinic acid (PSA), aniline 2-sulfinic acid (ANSA) (Figure 10) and TAHS (Figure 5) were also able to reduce lactate dehydrogenase and glutamate release, due to an increase in the GABAergic transmission caused by a reduction in the GABA metabolism [51,52].

## 5. Anti-Alcohol Activity

Both taurine and ethanol exert positive allosteric modulatory effects on neuronal ligand-gated chloride channels (GABA A and glycine receptors) as well as inhibitory effects on other ligand- and voltage-gated cation channels (NMDA and Ca^2+^ channels). Behavioral evidence suggests that taurine can alter the locomotor stimulatory, sedating, and motivational effects of ethanol in a strongly dose-dependent manner [53].

**Figure 11:**
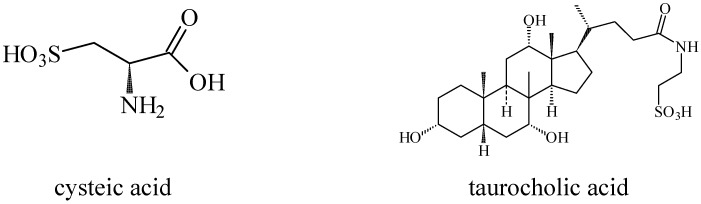
Structures of cysteic acid and taurocholic acid.

Research involving taurine analogues and ethanol intake had already been conducted by Messiha *et al.*, in which a comparison was made between some taurine precursors and metabolites. It was found that cysteic acid and taurocholic acid (Figure 11) had the best effect in the reduction of ethanol intake. The former enhanced the ethanol-induced sleep time and the latter reduced the onset of sleep [54].

**Figure 12:**
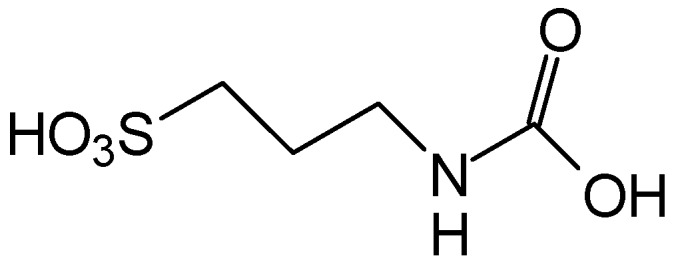
Structure of acamprosate.

The most successful derivative of taurine for alcohol abuse use is the acetylhomotaurine derivative named acamprosate (calcium acetylhomotaurinate, Figure 12). It can reduce ethanol self-administration and drinking relapses in both animals and humans. Taken together, these data suggest that the endogenous taurine system may be an important modulator of the effects of ethanol on the nervous system, and may represent a novel therapeutic avenue for the development of medications to treat alcohol abuse and alcoholism [53]. Indeed, in 2004, acamprosate (Campral™) was approved by the FDA for use in treating alcohol dependence. In a previous clinical trial report, acamprosate was also seen to be benefitial in alcohol-dependent individuals with bipolar disorder [44].

Surprisingly a report by Dzirkale showed an anti-ethanol effect, reducing the ethanol sleeping time with low doses with tauropyrone, a neuroportector compound described before, devoid of anticonvulsant activity [55].

## 6. Retina Protector

**Figure 13:**
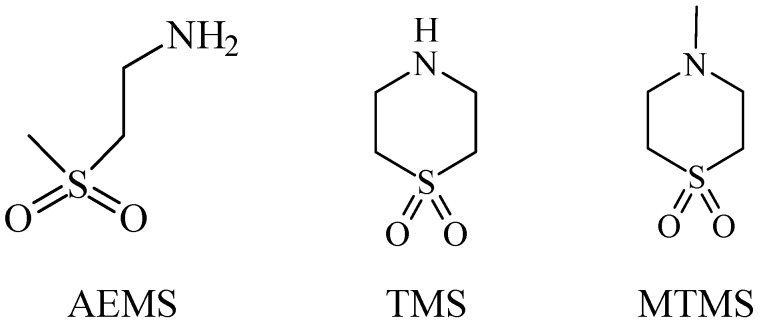
Structures of 2-aminoethylmethylsulfone (AEMS), thiomorpholine 1,1-dioxide (TMS) and *N*-methylthiomorpholine 1,1-dioxide (MTMS).

In normal organisms the retina possesses high levels of taurine, The greatest concentration of taurine is found in the photoreceptor cell layer of the retina. Taurine deficiency induces abnormal bipolar cell plasticity and induces retinal ganglion cells loss in mice [14]. Three derivatives: 2-aminoethylmethylsulfone (AEMS), thiomorpholine 1,1-dioxide (TMS) and *N*-methyl- thiomorpholine 1,1-dioxide (MTMS) (Figure 13) were shown to be the most promising compounds obtained. They showed more potency than other cyclic compounds such as CAHS and TAHS. They act by stimulating ATP-dependent Ca^2+^ uptake [56].

## 7. Anticancer

**Figure 14:**
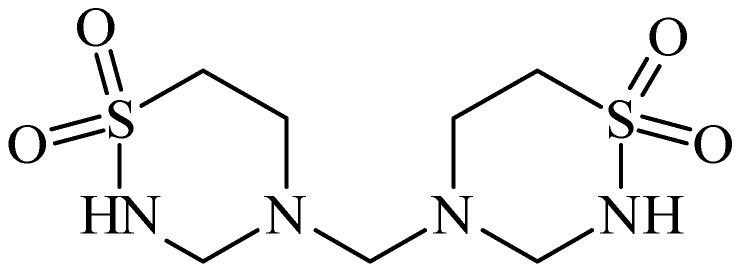
Structure of taurolidine.

Taurolidine (TRD, Figure 14) is a taurine derivative that was first described as an anti-bacterial substance, with anti-cancer properties due its pro-apoptotic actions [57,58]. It induces apoptosis through the appearance of cytocrome C, which is normally in the mitocondria, in the cytoplasm, and stimulating the activation of procaspases 8 and 9. Furthermore, taurolidine is known to increase the level of the protein Bax, decreasing the levels of Bcl-2, Mcl-1 and survivin. TRD has shown encouraging clinical results after intravenous administration in patients with gastrointestinal malignancies and tumors of the central nervous system [59]. Art and co-workers showed severe hepatotoxicity with taurolidine and the failure to inhibit primary osteosarcoma tumor growth in mice. However, more studies are needed to balance its benefits and toxicity in cancer treatment [60].

## 8. CLogP of the Compounds

Log P is a coefficient partition parameter used to measure the lipophilicity of the compounds and it can be used predict the membrane passive diffusion by drugs. We calculated Log P (CLogP) values using ChemBioDraw Ultra, version 12.0, Cambridge Software [61], of all above taurine derivative obtained for the treatment of CNS diseases. Table 2 shows the results. All the compounds presented ClogP values higher than that obtained for taurine (−4.7936), suggesting an increase of the lipophilicity. Positive values suggest passive diffusion across the membrane (BBB) and compounds with negative values may cross the membrane using a transport system (GABA or TAU). The results don’t show the correlation of values for CLogP and biological activity.

**Table 2:** CLogP * values of the derivatives of taurine with CNS activity.

Chemical Structures	Clog P	Pharmacological activities	References
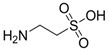	−4.7936	Physiological aminoacid taurine osmorregulator, neuromodulator, neuroprotector	
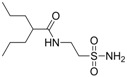	1.0880	Anticonvulsant	[35]
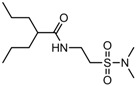	2.0546	Anticonvulsant	[35]
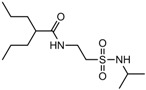	2.5066	Anticonvulsant	[35]
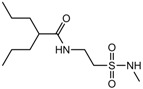	1.6686	Anticonvulsant	[35]
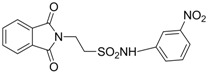	2.5711	Anticonvulsant	[33]
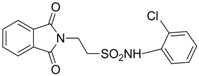	2.7571	Anticonvulsant	[33]
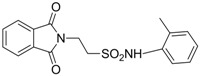	2.3471	Anticonvulsant	[33]
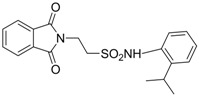	3.0251	Anticonvulsant	[33]
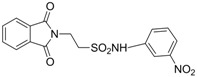	2.5711	Anticonvulsant.	[33]
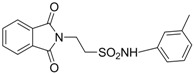	2.9071	Anticonvulsant	[33]
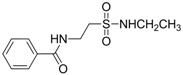	0.9486	Anticonvulsant	[37]
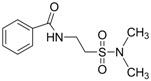	0.8056	Anticonvulsant	[37]
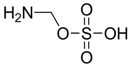	−3.6202	Hyperthermia by GABA antogonism	[38]
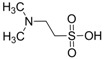	−3.8958	Hyperthermia by GABA antogonism	[38]
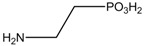	−3.9796	Hyperthermia by GABA antogonism	[38]
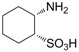	−3.3916	Hyperthermia by GABA antogonism	[39]
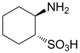	−3.3916	Hyperthermia by GABA antogonism	[39]
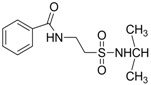	1.2576	Anticonvulsant	[37]
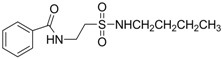	2,0066	Anticonvulsant	[37]
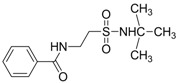	1.6566	Anticonvulsant	[37]
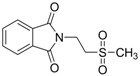	0.2834	Anticonvulsant	[37]
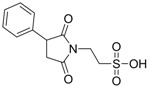	0.8374	Anticonvulsant	[37]
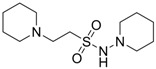	2.1490	Anticonvulsant	[37]
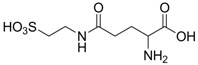	−4.7068	Alzheimer’s prevention and neuroprotection	[41,42]
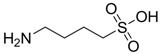	−4.4994	Alzheimer’s prevention and neuroprotection	[42,43,44]
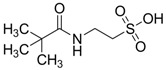	−1.3964	Alzheimer’s prevention and neuroprotection	[44,45,46]
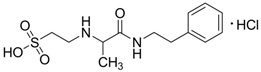	−2.8288	Alzheimer’s prevention and neuroprotection	[47,48,49,50]
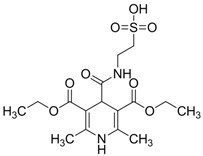	0.5202	Alzheimer’s and Parkinson’s disease prevention and neuroprotection	[51,52]
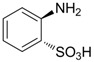	−1.8720	Alzheimer’s and Parkinson’s disease prevention and neuroprotection	[51,52]
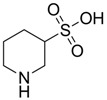	−3.3915	Alzheimer’s and Parkinson’s disease prevention and neuroprotection	[51,52]
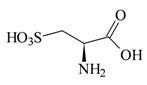	−4.471	Anti-alcohol activity	[53,54]
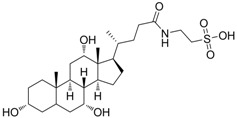	−0.7994	Anti-alcohol activity.	[53,54]
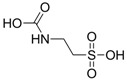	−3.0638	Anti-alcohol activity	[55]
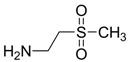	−1.6418	Retina protector	[14,56]
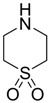	−1.3740	Retina protector	[14,56]
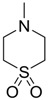	−0.7990	Retina protector	[14,56]
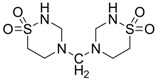	−0.0866	Anticancer	[57,58,59,60]

* ChemBiodraw Ultra 12.0, Cambridge Software, 2010 [61]

## 9. Conclusions

The design of new drugs is a challenging field, especially for CNS diseases. Firstly, due the pathophysiology of the diseases, most of them are not totally understood or do not occur in a specific area of the brain or are caused by more than one neurotransmitter and so on. Secondly, because of the properties of the compounds and their activities: How to be selective without side effects? How to block an excitatory brain activity without depressing the brain in non-injured areas? How to treat CNS while maintaining the safety of the brain? 

Taurine is an example of an essential amino acid that can be used as a strategy to develop new CNS drugs. Because of taurine’s neuroprotective effects, besides the ability to prevent seizures, it is a good motivation to develop new anticonvulsant and others derivatives which could mimic the actions of taurine. Using the knowledge about the essential biological amino acids and in addition to all the molecular modification tools such as bioisosterism, hybridization, prodrug design, and also with the medicinal chemistry researcher’s intuition it may be a simple strategy and alternative with quick and good response when specific targets are not known.
